# Frequency-dependent selection drives HBeAg seroconversion in chronic hepatitis B virus infection

**DOI:** 10.1093/emph/eot023

**Published:** 2013-12-13

**Authors:** Brook G. Warner, William G.H. Abbott, Allen G. Rodrigo

**Affiliations:** ^1^Bioinformatics Institute, University of Auckland, Private Bag 92-019, Auckland, New Zealand; ^2^The New Zealand Liver Transplant Unit, Auckland City Hospital, Private Bag 92-024, Auckland, New Zealand; ^3^Biology Department, Duke University, 125 Science Drive, Durham, NC 27708, USA

**Keywords:** HBeAg seroconversion, hepatitis B virus, HBeAg, frequency dependent selection

## Abstract

HBeAg seroconversion is an important stage in the evolution of a chronic hepatitis B virus (HBV) infection that usually leads to control of viral replication and a reduced risk for liver cirrhosis and cancer. Since current therapies for the HBV-associated liver inflammation that is known as chronic hepatitis B (CHB). Rarely induce permanent HBeAg seroconversion, there is a need to understand the mechanisms responsible for the purpose of identifying new therapeutic targets. Currently, the most widely accepted hypothesis is that the patient’s humoral and cellular immune responses to the HBV initiate HBeAg seroconversion. Although we accept that this hypothesis cannot be excluded, we propose an alternative that is consistent with published data on HBeAg seroconversion. We postulate, as others have, that the HBeAg suppresses the immune response to the HBV. However, production of the HBeAg incurs a metabolic cost to the hepatocyte which reduces the replicative capacity of the virus. Consequently, HBeAg-negative viruses replicate faster than HBeAg-positive viruses. HBeAg-negative variants arise *de novo*; and when their frequency in the population is low they have a replicative advantage. However, they also benefit from the immunosuppressive effects of the HBeAg-positive viruses in the population. As HBeAg-negative variants increase in frequency and HBeAg levels fall, the immune system recognizes the HBV, and HBeAg seroconversion occurs as a consequence of frequency-dependent selection acting on HBeAg-negative variants. This hypothesis explains the wide inter-individual variation in age of seroconversion, the increased rate of seroconversion during anti-viral treatment and the phenomena of both spontaneous and post-treatment HBeAg reversions (in which patients cycle between the HBeAg-positive and negative phases of their infection).

## INTRODUCTION

Negative frequency-dependent selection (NFDS) occurs when the fitness of a phenotype in a population is inversely proportional to its frequency in that population. This implies that opposing forces acting for and against selection of the phenotype are dominant at either low or high phenotype frequencies, respectively, and provides a mechanism to account for fluctuations in the frequency of a phenotype over time. In patients with a chronic hepatitis B virus (HBV) infection, fluctuations in frequency of mutant viruses that are unable to make the immunosuppressive HBeAg are associated with clinically significant changes in immune reactivity to the virus. The phenomenon of NFDS provides a framework for generating hypotheses about the mechanisms that might drive these fluctuations, and provide insights that might lead to new therapies for the HBV-associated liver inflammation known as chronic hepatitis B (CHB).

The soluble hepatitis B e-antigen (HBeAg) is encoded within the C open reading frame of the HBV genome. Translation of the full C open reading frame produces the p25 polypeptide, from which the secreted HBeAg is produced by post-translational modification. This occurs in two steps. The first 19 amino acids direct p25 to the secretory apparatus of the cell where it is cleaved to produce the p22 polypeptide as it enters the endoplasmic reticulum. The p22 polypeptide either undergoes C-terminal cleavage in the Golgi apparatus to produce the secreted HBeAg, or is released back into the cytoplasm. High levels of serum HBeAg are found in the early ‘immune tolerant’ stage of a chronic HBV infection [1], and it is thought that precore proteins contribute to the development of chronic HBV infections by suppressing innate, humoral and cellular immune responses to the HBV [2]. Immune tolerance to the HBV is eventually lost in most patients, and this loss is generally associated with a decrease in the serum levels of HBV-DNA and HBeAg, the appearance of anti-HBeAg antibodies in serum and evidence of liver inflammation. This process is known as HBeAg seroconversion, and it is usually preceded by the selection of genetic variants with mutations in the core gene promoter and the precore region of the C open reading frame that either reduce or abrogate HBeAg transcription or translation [3, 4]. We will refer to these as HBeAg-negative mutant viruses. Acquired humoral and cellular immunity to HBV antigens also becomes detectable in the peripheral blood and the liver at this time [5, 6], and CD8+ T-cell mediated suppression of viral replication associated with cessation of liver inflammation results in the inactive, healthy carrier state [1].

Between 10% and 20% of patients do not permanently suppress viral replication at the time immune tolerance is lost, and develop a form of chronic liver inflammation known as CHB. CHB is associated with high risks of liver cirrhosis and liver cancer [1]. The primary goals of antiviral treatments for CHB are suppression of viral replication and induction of HBeAg seroconversion. Suppression of viral replication results in cessation of liver inflammation and a decreased risk of serious sequelae. Permanent induction of HBeAg seroconversion should result in control of viral replication by the patient’s immune system, allowing cessation of antiviral treatment. Unfortunately, most patients do not achieve HBeAg seroconversion on treatment; and those that do usually relapse when treatment is stopped [1, 7]. Consequently, these treatments need to be life-long, which is inconvenient and expensive. New therapeutic targets need to be identified, which will require an understanding of the mechanisms that result in HBeAg seroconversion. Although it is currently hypothesized that innate and acquired immune responses to the HBV are responsible for HBeAg seroconversion [8], we are presenting an alternative hypothesis which states that it is the result of a process that manifests as NFDS of HBeAg-negative mutant viruses.

## NEGATIVE FREQUENCY-DEPENDENT SELECTION AND GENETIC DRIFT CAN ACCOUNT FOR THE CHANGES IN SERUM LEVELS OF HBeAg-NEGATIVE MUTANT VIRIONS WITH TIME

**Figure 1.**
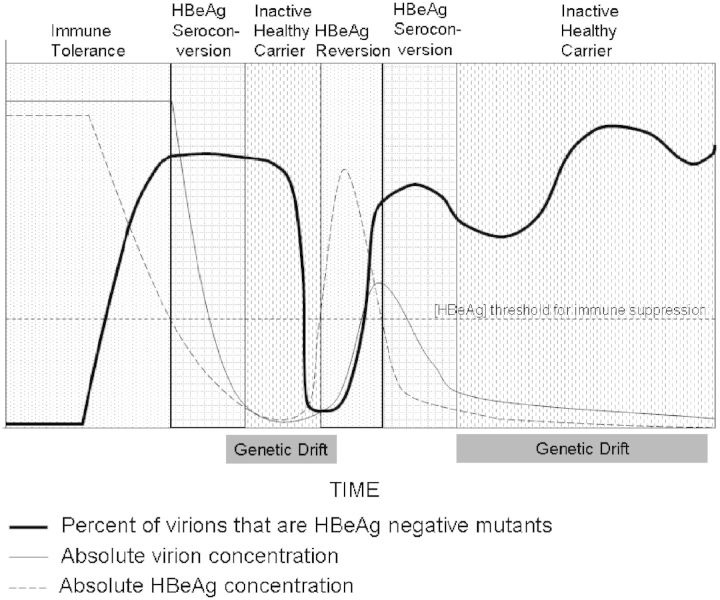
This figure demonstrates how frequency dependent selection and genetic drift control the percentage of viruses in serum that are HBeAg-negative mutants. The first panel shows the immune tolerant stage of the infection characterized initially by high levels of wild-type virus (thin straight line) and HBeAg (broken line) in the serum. The frequency of HBeAg-negative mutants is initially very low, but at some point starts to increase in frequency due to unknown mechanisms that favor their selection relative to wild-type viruses. As the frequency of HBeAg-mutant viruses increases, then the frequency of wild-type viruses falls, together with the level of serum HBeAg. When the level of HBeAg falls to a level that no longer suppresses the immune system, then viral replication will be suppressed and the level of serum and hepatic virions will decrease, and the patient will enter the inactive healthy carrier stage of the infection. A low HBV population size will also result in an increase in genetic drift. This is a random process, and in the third panel we are showing the outcome in which there is an increase in wild-type virions that secrete the HBeAg, accompanied by a reciprocal sharp fall in the frequency of HBeAg-negative mutants. When the level of HBeAg reaches a point that the immune response to the HBV is suppressed, the viral titers will start to rise again. This is known as HBeAg reversion and the patient re-enters the immune tolerant stage of infection. The mechanisms that preferentially select for replication of HBeAg-mutants are re-activated, and the process of HBeAg seroconversion is re-initiated. In the final panel, we show the situation where genetic drift does not result in a large enough increase in wild-type virus replication to cause HBeAg reversion. In this case the patient remains in the inactive healthy carrier stage of infection, with some fluctuation in the relative frequency of wild-type and HBeAg-negative mutant viruses

The clinical observation that some subjects fluctuate between the HBeAg-positive and HBeAg-negative states (known as HBeAg reversion [1]) raises the possibility that there are forces that select for and against replication of HBeAg-negative mutant relative to wild-type virions at different times. We hypothesize that wild-type HBV have lower replication efficiencies within hepatocytes that are producing the immunosuppressive HBeAg; and further propose that NFDS in combination with genetic drift accounts not only for HBeAg reversion but also for the fluctuations in serum levels of HBeAg-negative mutant virions that occur over time in patients with chronic HBV infections (Fig. 1). In other words, HBeAg-negative mutants behave as if they have a replication advantage over wild-type viruses when mutant levels are low; but this advantage decreases as levels of the mutant virus increase. The influence of NFDS may be enhanced or opposed by genetic drift; defined as stochastic fluctuations in the relative frequencies of genetic variants (such as the wild-type virus and the HBeAg-negative mutant viruses) within a population due to the amplification of sampling effects that occurs when there is a small effective population size [9]. It is important to note that effective population size is not equivalent to the number of individuals in a population. Effective population size is the number of replicating virions in an ‘ideal’ population that would have the same level of genetic drift as the non-ideal population being studied. For example, if there was a high level of variability in the number of viruses produced/day by each cccDNA mini-chromosome in a liver, so that the majority of serum virions were produced from a small fraction of the total cccDNA pool, then the effective population size would be small and there would be a high rate of genetic drift in the population, even in the presence of the high viral loads found in HBeAg-positive subjects. Since genetic drift tends to favor fixation of the most common species within a population, this would usually oppose an increase in frequency of HBeAg-negative mutants in HBeAg-positive subjects. If nucleot(s)ide analog therapy reduced viral replication so that the number of virions produced/day by each cccDNA was equalized, then the effective population size would increase, genetic drift would decrease and an increased frequency of HBeAg-negative mutants with a replication advantage could emerge. This might contribute to the increased rate of HBeAg seroconversion that occurs in patients on treatment.

We propose that HBeAg-negative mutants arise because of random transcription errors [3]. When the frequency of HBeAg-negative mutant viruses in the liver is low (as in the early immune tolerant stage of a chronic HBV infection), then the cells containing HBeAg-negative mutants will produce a relatively larger number of virions than cells with wild-type virus. This may be due to a replicative advantage of mutant relative to wild-type viruses [10, 11] as a result of energy savings due to either abrogated translation of the HBeAg or reduced transcription of precore mRNA [12]. The percentage of HBeAg-negative mutants in serum would then start to increase at a rate determined by the balance between the size of the fitness advantage of the HBeAg-negative haplotype, the effective population size and the availability of new replication spaces in the liver. Subjects with a low effective population size would continue to have a low frequency of mutant viruses in serum. However, if the effective population size was high there would be an inexorable increase in frequency of HBeAg-negative mutant viruses in the immune tolerant phase of the infection; because even a small selective advantage would have an impact on the success of HBeAg-negative variants. Eventually, the frequency of hepatocytes secreting the HBeAg would fall and be accompanied by a decrease in serum HBeAg concentrations. Loss of the HBeAg is believed to be associated with increased CD8+ T-cell immunity to the HBV [5, 13], and suppression of replication of both wild-type and mutant virions. At this point there is HBeAg seroconversion, and a concomitant decrease in the viral load. When the virus population drops to very low levels, the replicative advantage of HBeAg-negative variants over HBeAg-positive wild-type viruses may be swamped by the stochastic effects of genetic drift. This may lead, in some patients, to an increase in the frequency of wild type relative to HBeAg-mutant virions and increased HBeAg secretion. We postulate that this may be the mechanism for HBeAg reversion. Should this occur, we expect that the cycle would repeat itself: as the total viral population increases in size, HBeAg-negative variants that remain in the population or that emerge *de novo* through mutation increase in frequency because of their replicative advantage. This in turn leads to a reduced production of HBeAg and is followed by a renewed immune response coupled with decreasing virus loads. It is possible to imagine several cycles of HBeAg reversion driven by alternating NFDS and genetic drift. Alternatively, continued dominance of HBeAg-negative mutant virions associated with the development of host immune responses that were strong enough to maintain low levels of HBV replication would result in permanent development of the inactive, healthy carrier state.

## ASSUMPTIONS OF THE NEGATIVE FREQUENCY-DEPENDENT SELECTION HYPOTHESIS

The first assumption of the NFDS hypothesis is that HBeAg-negative virions have a replication advantage over wild-type virions that are able to produce the HBeAg; and a replicative advantage of HBeAg-negative precore mutants has been reported by some [10, 11] but not all [14] authors who have investigated this issue. This variability in results may have occurred because the size of any replicative advantage that is sufficient to initiate the process of HBeAg seroconversion may be too small to detect by experimental systems that compare replicative rates in parallel cultures. Resolution of this issue might be obtained using a competitive assay system in which cultured cells are infected with different ratios of wild-type to mutant virions (Table 1).

**Table 1.** Summary of hypotheses to test the assumptions and predictions of the NFDS model

Hypothesis	Possible experimental test	NFDS model supported if …
Increased replication rate of HBeAg-negative versus HBeAg-positive virions.	Measure replication rates *in vitro*, with direct competition between HBeAg-positive and HBeAg-negative virions.	HBeAg-negative virions outgrow HBeAg-positive virions.
Reversible suppression of HBV-specific CD8+ T cells by precore proteins.	Currently difficult to do, but will probably require cultures of CD8+ T cells *in vitro* or in animal models.	HBV core peptide-specific CD8+ T cells recover function after removal of exposure to precore proteins.
HBeAg reversion caused by genetic drift (i.e. patients with low minichromosome effective population size).	Identify patients who have become HBeAg-negative during nucleos(t)ide analog therapy for CHB. Measure neutral selection on the HBV genome at baseline and while they are still HBeAg-negative.	HBeAg reversion associated with a high frequency of HBV codons under neutral selection.
Genetic drift increases as viral load declines	Measure neutral selection in serial HBV-DNA samples from HBeAg-negative patients with declining viral loads.	Increased frequency of codons under neutral selection at low viral load
Decreased measured strength of positive selection as viral load declines.	Measure positive selection in serial HBV-DNA samples from HBeAg-negative patients with declining viral loads.	Decreased omega (ω) at positively selected codons at low viral load

A second assumption required by the NFDS hypothesis is that precore proteins are immunosuppressive, and essential for initiation and maintenance of the immune tolerant phase of a chronic HBV infection. There is clinical evidence consistent with this assumption. For example, the incidence of chronic HBV infection is higher in the infants of HBeAg-positive relative to HBeAg-negative mothers, although this may be due to higher levels of serum HBV in HBeAg-positive mothers [15]. The corollary of this belief is that a chronic infection cannot be induced by infection with an HBeAg-negative mutant virus. This is supported by data from small numbers of chimpanzees [16]. In laboratory studies, serum HBeAg has been shown to elicit CD8+ T-cell tolerance to the core antigen in mice [17], and cytoplasmic, precore proteins impair innate signaling in cell lines [2]. However, the molecular mechanisms that might be responsible for precore protein-mediated immunosuppression are still poorly understood.

Third, the NFDS hypothesis assumes that precore protein-mediated suppression of anti-HBV immunity is at least partly reversible when precore protein levels decrease. At a clinical level there is an association between loss of serum HBeAg and increased anti-HBe levels [1], increased CD8+ T-cell activity against the HBV [5, 13] and decreased viral replication [1]. Increased genetic diversity in the HBV core gene has also been associated with decreased HBV-DNA and HBeAg levels in HBeAg-positive subjects [18].

It is also possible that serum HBeAg has no function in humans besides induction of vertical transmission; and that the contribution of precore proteins to maintenance of the immune tolerant phase of a chronic HBV infection is entirely due to suppression of cytosolic innate immunity within hepatocytes [2]. In this case the decrease in serum HBeAg levels would be a marker for reduced immunosuppression rather than part of the mechanism. If precore proteins do suppress intracellular innate immunity [2], then loss of cytosolic precore proteins as a result of NFDS could result in increased type I interferon and inflammatory cytokine production [19] by any hepatocytes that were then able to respond to HBV pathogen-associated molecular patterns. Under this scenario, both cytosolic and cellular immune responses could then contribute to the gradual increase in positive selection pressure on the HBV core gene that has been reported to precede HBeAg seroconversion [18]. However, in the absence of hepatocyte death and uptake by DCs it is unclear how any activated DCs would present the correct peptides to CD8+ T cells in lymph nodes. It is also unclear how any activated DCs that did present the correct peptides would reverse exhaustion and/or anergy of HBV-specific CD8+ T cells. Identification of the mechanisms whereby loss of either or both of cytosolic precore proteins and serum HBeAg contribute to the resurgence of CD8+ T-cell function would be of great value for development of new therapies.

Fourth, if NFDS and genetic drift are entirely responsible for initiation of HBeAg seroconversion without input from the host immune system, then there must be host factors that influence the magnitude of the replicative advantage of HBeAg-negative mutants and/or the effective population size of cccDNA mini-chromosomes. This is necessary to account for both inter-individual variations in the age at which spontaneous seroconversion occurs and inter-individual variation in the response to antiviral therapy. Clinical parameters that may explain some of this variance include age of initial HBV infection [1], age of puberty [20] and host ethnicity [1].

## CONSEQUENCES AND PREDICTIONS OF THE FREQUENCY-DEPENDENT SELECTION HYPOTHESIS

The first prediction of this hypothesis is that the fall in serum HBeAg levels will precede both the fall in HBV-DNA levels and HBeAg seroconversion. Although low HBeAg levels precede seroconversion [18], we are not aware of any longitudinal data in immune tolerant subjects that allows the timing of the decreases in HBeAg and HBV-DNA levels to be compared. The associations between low HBeAg levels and both low HBV-DNA levels and increased genetic diversity [18], and the finding that high precore mutation levels predict HBeAg seroconversion on treatment [21] are consistent with the prediction that low HBeAg levels precede and contribute mechanistically to seroconversion (Table 1).

The second prediction of the hypothesis is that there should be more positive selection pressure on the HBV in HBeAg-negative than HBeAg-positive subjects. Positive selection pressure occurs when immune mechanisms that suppress replication of the HBV force the selection of virions that contain amino acid substitutions (escape mutations) in the immune epitopes they recognize. Amino acids under positive selection can be identified in cloned viral DNA by finding a high ratio of non-synonymous to synonymous nucleotide mutations in the codons that encode them. An increased frequency of positively selected mutations has been shown in the core gene of the HBV from HBeAg-negative patients [13].

A third prediction of the hypothesis is that HBeAg-negative mutant viruses will be detectable in the serum of patients who are classified as being in the HBeAg-positive phase of their infection, and the frequency of these mutant viruses will increase with time. Published data, in which serial measurements of the frequency of mutant viruses have been made in HBeAg-positive subjects, strongly supports both these predictions [3, 4, 18].

A fourth prediction of the hypothesis is that there will be increased evidence of genetic drift in the viral genome as the viral load declines. Testing this prediction would require sequencing of cloned HBV genomes obtained from serial serum samples of patients with an HBeAg-negative chronic HBV infection, followed by analyses that test for neutral evolution. The increase in drift may also exceed the influence of the selective effects that were initially responsible for the increase in frequency of HBeAg-negative mutants relative to wild-type viruses. This would result in fluctuations in the relative frequencies of HBeAg-negative mutant and wild-type viruses in serum of HBeAg-negative patients, as has been reported by Nie *et al.* [4].

A fifth prediction is that the increase in genetic drift that occurs as viral load declines will result in a decrease in the measured strength of positive selection pressure on the virus, even though the immune mechanisms suppressing viral replication remain unchanged. This is because stochastic differences in viral reproduction fitness can overwhelm the replicative advantage of fitter viruses at low population sizes. We are not aware of any published data that addresses this prediction.

## ALTERNATIVE HYPOTHESES FOR INITIATION OF HBeAg SEROCONVERSION

If HBeAg-negative mutant virions do not have a replicative advantage relative to wild-type virions then alternative hypotheses for initiation of HBeAg seroconversion need to be considered (Table 2). A gradual increase in genetic diversity in the HBV core gene has been reported to occur several years before the completion of HBeAg seroconversion [18], which raises the possibility that an immune response to core protein epitopes could be important; and both core-specific T-helper cell activity [22] and anti-HBe antibodies [23] have been detected before HBeAg seroconversion has occurred. There are also a number of published associations between humoral and cellular responses to the HBV and HBeAg seroconversion [8]. Both spontaneous and treatment-induced seroconversions are associated with a high frequency of CXCR5+ve, CD4+ T cells that can boost anti-HBe production using an IL-21-related mechanism [24]. A high rate of spontaneous HBeAg seroconversion has been predicted both by polymorphisms in the interleukin (IL)-10 and IL-12 promoters [25] and by increased serum IL-10 and IL-12 concentrations [25]. A number of non-immunological variables that could conceivably be associated with anti-viral immune activity also predict high rates of spontaneous HBeAg seroconversion. These include high serum alanine transaminase (ALT) levels [26], high mutation rates in all open reading frames [3] and increased viral diversity [27]. Immune variables and immune-associated variables also predict HBeAg seroconversion on anti-viral therapy. Increased serum IL-12 concentrations precede or coincide with seroconversion [28] and high serum IL-21 levels after 12 weeks of therapy predict seroconversion [29]. As with spontaneous seroconversion, treatment-induced HBeAg seroconversion is associated with high baseline ALT levels [30], low baseline HBV DNA levels [30] and increased viral diversity [27].

**Table 2.** Summary of hypotheses to support an immune basis for selection of HBeAg-negative mutants

Hypothesis	Suggested experimental test	Immunity model supported if …
Anti-HBe antibodies suppress viral replication.	Look for anti-HBe antibodies within hepatocytes or liver cell lines.	Intracellular anti-HBe demonstrated and associated with reduced HBV replication. Anti-HBe/HBeAg complexes activate cytosolic innate immunity. Positive selection pressure on HBV core gene demonstrated within anti-HBe binding site.
CD8+ T cells suppress viral replication in HBeAg-positive subjects.	Simultaneous measurements of peptide-specific CD8+ T-cell activity and precore mutant virions.	Positive correlation between pre-core mutant virion levels and CD8+ T-cell activity.
NK cells suppress viral replication.	Currently difficult to do.	
Cytosolic innate immunity suppresses viral replication after detecting precore proteins/mRNA.	Transfect mammalian cell lines with precore genes.	Expression of precore genes associated with activation of interferon-stimulated genes.

These data raise the issue of whether these associations arise because humoral and cellular immune responses initiate seroconversion or whether they are the result of seroconversion. Any immune response that initiated the selection of HBeAg-negative mutant virions would need to suppress replication of wild-type virions following recognition of a precore protein component. However, it is difficult to identify either a humoral or cellular immune mechanism that could satisfy this criterion in HBeAg-positive subjects. There is no evidence that CD4+ T cells exert selection pressure on the HBV; and CD8+ T cells are unlikely to exert direct selection pressure on precore and core promoter loci, as these mutations are not HLA class I restricted. Data in mice [31] raised the possibility that hepatocytes expressing both the HBcAg and HBeAg are more susceptible to lysis by CD8+ T-cell responses to their shared epitopes than hepatocytes expressing HBcAg only. However, this does not necessarily apply to humans, as there is no evidence that functional, HBV-specific CD8+ T cells exist in HBeAg-positive subjects [5, 32]; and the usual method of viral escape from CD8+ T-cell recognition is the evolution of amino acid substitutions within the peptides presented by HLA class I [13, 33]. These substitutions in HBV C open reading frame peptides are rarely observed in virions from HBeAg-positive subjects [13]. An effect of natural killer (NK) cells is more difficult to exclude, as NK cells may be able to recognize specific viral sequences in association with HLA class I [34]. However, there is no current evidence that NK cells respond to the HBeAg. Although T-cell dependent anti-HBe antibodies [23] have been reported in most immune tolerant patients, there is no known mechanism whereby they can suppress viral replication within hepatocytes. Thus, any contribution these humoral and cellular responses make to HBeAg seroconversion could be secondary to some other primary cause of decreased precore protein production.

There is very little data addressing the possibility that intracellular innate immunity might initiate HBeAg seroconversion. Another alternative to the NFDS hypothesis is that innate molecules detect precore mRNA or precore proteins within hepatocytes [35], resulting in suppression of viral replication by interferon-stimulated genes and forcing selection of HBeAg-negative mutant viruses within those hepatocytes. In other words, HBeAg-negative mutants would be selected because HBV replication was impossible in a hepatocyte that was making HBeAg. If it is correct that innate recognition of either a pathogen or a damaged host structure is necessary for an acquired immune response to be generated [2], then intracellular innate recognition of a precore gene product would account for both the initial HBeAg loss as well as the development of the acquired immune responses that eventually control all HBV replication; even though the innate molecular mechanisms that recognize HBV components have yet to be identified within either hepatocytes or the cellular immune system. Of particular interest is the possibility that if there is a system for suppressing HBV replication following detection of the HBeAg or its precursors in hepatocytes, then this system might need to be induced by environmental factors. A requirement for an environmental factor to induce seroconversion would account for neonates and young children being especially susceptible to chronic HBV infections [1] and for the increase in HBeAg seroconversion rates that occurs with age [1]. Identification of such an environmental factor would be of value in designing new therapies for CHB.

## SUMMARY

Currently, NFDS of HBeAg-negative mutant viruses and genetic drift can account for all published data describing the changes in frequency of both wild-type and HBeAg-negative mutant virions over time in humans with a chronic HBV infection; provided the assumptions of a replicative advantage for mutant virions and of a reversible, immunosuppressive function for precore proteins are true. Prospective measurements of the influence of genetic drift and selection pressure on the evolution of a chronic HBV infection would provide further support for the hypothesis, as would stronger evidence that HBeAg-negative mutant virions have a replication advantage over wild-type virions (see Table 1). There are two significant consequences of this hypothesis being correct. First, immune-based therapies are unlikely to initiate HBeAg seroconversion. Drugs that increase the number of mutant viruses available for selection may become a therapeutic option [36], and strategies that increase the effective population size of the virus might be useful. Second, either serum HBeAg and/or cytoplasmic precore proteins must be responsible for reversible suppression of the CD8+ T-cell response to HBV peptides. Identification of the molecular mechanisms responsible would be of value for developing immunotherapies for CHB.

However, alternative hypotheses stating that the host immune system is responsible for HBeAg seroconversion cannot currently be excluded. We have highlighted a number of limitations to the hypothesis that humoral or cellular immune responses to the HBV are responsible for initiating HBeAg seroconversion. As an alternative, we suggest that seroconversion may be caused by an inducible, cytosolic, innate immune mechanism that abrogates HBV replication in a hepatocyte that is producing precore proteins. The attraction of this hypothesis is that identifying such an inducible mechanism would provide targets for novel therapies.

**Conflict of interest**: None declared.
